# Intermediate‐Phenotype ABCA3 Deficiency Caused by Compound Heterozygous Variants Presenting as Persistent Respiratory Failure and Systemic Hypertension Since Birth in a Term Infant: A Case Report

**DOI:** 10.1002/ccr3.73587

**Published:** 2026-09-28

**Authors:** Ammir Abuzahra, Mohamad Abu Mayalah, Mahmoud Abdelrazzaq Abu Mayaleh, Amani Daabes, Rafad Y. Dweik, Abdelrazzaq Abu Mayaleh

**Affiliations:** ^1^ College of Medicine, Hebron University Hebron Palestine; ^2^ Department of Pediatric Pediatric Resident at RCHS Hospital Hebron Palestine; ^3^ College of Medicine and Health Sciences Hebron University Hebron Palestine; ^4^ Scientific Research and Medical Development Center PRCS Hospital Hebron Palestine; ^5^ Pediatric Pulmonology RCHS Hospital Hebron Palestine; ^6^ Department of Pediatric & Neonatal Intensive Care Unit (PICU/NICU) Palestine Red Crescent Society (PRCS) Specialized Hospital Hebron Palestine; ^7^ Scientific Research and Medical Development Unit Palestine Red Crescent Society (PRCS) Hebron Palestine; ^8^ Faculty of Medicine, Al‐Quds University Abu Dis Palestine

**Keywords:** ABCA3, childhood interstitial lung disease, hydroxychloroquine, infant, neonatal respiratory distress, rare disease, surfactant metabolism dysfunction, whole‐exome sequencing

## Abstract

ABCA3 deficiency should be suspected in term infants with persistent respiratory distress from birth. This case highlights how early whole‐exome sequencing established the diagnosis after extensive investigations, identified an intermediate phenotype caused by compound heterozygous variants, and guided targeted multidisciplinary management.

## Key Points

- Persistent respiratory distress from birth in a term infant, especially with repeated failure to wean respiratory support, should prompt early consideration of surfactant dysfunction disorders.
- ABCA3 compound heterozygosity involving one loss‐of‐function allele and one missense allele can produce an intermediate phenotype rather than immediately fatal neonatal disease.
- Imaging and bronchoscopy help exclude mimics, but genomic testing is the key confirmatory tool in suspected inherited surfactant disease.
- Pulmonary hypertension has been reported in ABCA3 deficiency, but systemic arterial hypertension should not automatically be attributed to the genetic disorder and requires an independent evaluation for secondary or treatment‐related causes.
- Outcome depends not only on pulmonary status but also on management of nutrition, aspiration, blood pressure, vascular access complications, and infection prevention.

**Key Clinical Message**

Persistent respiratory distress from birth in term infants should prompt early consideration of ABCA3 deficiency. Whole‐exome sequencing can establish the diagnosis, shorten diagnostic delay, guide management, and identify intermediate phenotypes caused by compound heterozygous variants.

## 1 Introduction

Pulmonary surfactant dysfunction disorders are a rare but important cause of persistent respiratory failure and chronic diffuse lung disease in infants. Among them, ABCA3‐related disease is especially relevant because ABCA3 encodes a lamellar‐body membrane lipid transporter that is essential for surfactant phospholipid handling, lamellar‐body integrity, and downstream processing of surfactant‐associated proteins. When ABCA3 function is severely impaired, the result may be profound neonatal respiratory failure; when partial function is retained, the phenotype may evolve as chronic oxygen dependence or infantile/childhood interstitial lung disease [1]. This wide clinical spectrum makes early recognition difficult, particularly when the infant is term‐born and repeatedly treated as bronchiolitis, pneumonia, or aspiration [2].

The present case is notable for five reasons. First, respiratory distress began at birth and persisted across several admissions. Second, the radiologic pattern was persistent yet non‐specific, prolonging the differential diagnosis. Third, the infant underwent extensive exclusion of common mimics, including reflux/aspiration, structural airway disease, primary ciliary dyskinesia, immunodeficiency, and cardiac disease before molecular confirmation was obtained. Fourth, whole‐exome sequencing identified a clinically meaningful compound heterozygous ABCA3 genotype comprising a novel frameshift allele and a missense allele previously reported in interstitial lung disease. Fifth, short‐term outpatient stabilization was achieved under a multidisciplinary regimen that combined supportive care with azithromycin, prednisone, and hydroxychloroquine.

## 2 Case Presentation

### 2.1 Patient Information

A 56‐day‐old female infant was transferred to the pediatric intensive care unit (PICU) for the management of escalating respiratory distress. The patient was born at 37 weeks of gestation via normal vaginal delivery with a birth weight of 2500 g to healthy, non‐consanguineous parents (a 23‐year‐old mother, gravida 1, para 1, abortus 0; and a 24‐year‐old father). The maternal, prenatal, and family histories were unremarkable, with normal prenatal ultrasound imaging and no history of maternal fever, premature rupture of membranes, or gestational diabetes.

The patient's neonatal medical history included a nine‐day admission to the neonatal intensive care unit (NICU) immediately following birth for tachypnea, oxygen desaturation, and retractions, managed as transient tachypnea of the newborn. At 11 days of age, she was readmitted to the NICU for eight days due to respiratory distress and left upper lobe opacification. She was treated with non‐invasive positive pressure ventilation (NIPPV) for three days and intravenous ampicillin and cefotaxime before being weaned to room air and discharged.

### 2.2 Presenting Concerns and Symptoms

Four days prior to the second admission, the infant developed a productive cough and rhinorrhea. She received outpatient treatment with hypertonic saline and albuterol nebulization, which provided minimal relief. Symptoms progressed over the subsequent 48 h to include severe difficulty breathing and poor oral intake. There was no history of fever, vomiting, diarrhea, cyanosis, or loss of consciousness. She was admitted to a local hospital with a presumptive diagnosis of acute bronchiolitis, received supplemental oxygen at 1 L/min via nasal cannula, nebulized therapies, and intravenous ampicillin and cefotaxime. Due to escalating respiratory distress and an increased oxygen requirement of 4 L/min, antibiotics were broadened to piperacillin‐tazobactam, intravenous hydrocortisone was administered, and she was transferred to the PICU.

### 2.3 Clinical Findings

Upon PICU admission, the patient was alert but in evident respiratory distress. Vital signs included a heart rate of 110 beats/min, a respiratory rate of 68 breaths/min, a blood pressure of 98/71 mmHg, and an oxygen saturation of 95% on 4 L/min via nasal cannula. Anthropometric measurements included a weight of 3950 g, length of 55 cm, and head circumference of 36.5 cm. Physical examination revealed prominent suprasternal and subcostal retractions, grunting, and globally decreased air entry without adventitious breath sounds. The anterior fontanelle was open and flat. Cardiovascular, abdominal, and neurological examinations were unremarkable; capillary refill time was less than two seconds, and the Moro reflex was active.

### 2.4 Timeline

The patient was born at term and was admitted to the neonatal intensive care unit (NICU) for 9 days because of tachypnea and oxygen desaturation. At 11 days of age, she was readmitted to the NICU for 8 days due to left upper lobe pulmonary infiltrates requiring noninvasive positive‐pressure ventilation (NIPPV). At 56 days of age, she developed acute respiratory failure and was admitted to the pediatric intensive care unit (PICU), where NIPPV and empirical antibiotic therapy were initiated. On hospital day 35, whole‐exome sequencing (WES) confirmed the underlying genetic diagnosis, and targeted therapy was subsequently initiated. At 3 months of age, she developed secondary systemic hypertension, necessitating treatment with a multidrug antihypertensive regimen. At 4 months of age, she underwent exploratory laparotomy with adhesiolysis and placement of an orojejunal feeding tube. At the most recent follow‐up at 5 months of age, she remained clinically stable on targeted therapy, with persistent baseline tachypnea managed in the outpatient setting. The complete chronological clinical course is summarized in Table 1.

**TABLE 1:** Chronologic timeline of respiratory deterioration, molecular diagnosis, major complications, surgery, and outpatient follow‐up.

Age or time point	Clinical event	Key findings and consequences
Birth (8/Dec/2025)	Term delivery at 37 weeks by normal vaginal delivery	Birth weight 2500 g; immediate NICU admission for tachypnea, desaturation, and retractions
Early neonatal period	First NICU stay	Managed as TTN‐like illness; echocardiography reportedly showed PFO and mild TR with preserved LV function
11 days	Second NICU admission	Respiratory distress with left upper‐lobe opacity/atelectatic change; NIPPV for 3 days; discharged after improvement
56 days	PICU admission	Productive cough, rhinorrhea, severe respiratory distress, and increasing oxygen requirement; admitted as bronchiolitis/rule‐out chest infection
Early PICU course	Escalated respiratory support	NIPPV‐RAM/non‐invasive ventilation was used intermittently; chest radiography showed hyperinflation with bilateral infiltrative change
Prolonged admission	Chest CT and repeat HRCT	Bilateral diffuse perihilar consolidation/air‐space opacities, sparing apices and basal regions; no meaningful interval change
Prolonged admission	Reflux and aspiration work‐up	The upper GI study showed Grade 3 reflux with aspiration and no gastric outlet obstruction
Prolonged admission	Airway/structural evaluation	Rigid bronchoscopy showed no H‐type fistula; endoscopy was grossly normal
Around hospital day 35	Molecular diagnosis	WES identified compound heterozygous ABCA3 likely pathogenic variants; targeted therapy initiated
Around 3 months	Secondary hypertension	Multiple high BP readings; echocardiography showed moderate LVH and EF 76%; renal CTA unremarkable
During prolonged admission	Vascular complication	Femoral‐line‐associated lower‐limb swelling; Doppler showed short‐segment DVT; enoxaparin started
4 months 3 days	Exploratory laparotomy	Adhesions released at the fourth part of the duodenum/proximal jejunum; orojejunal tube inserted
4 months 21 days	Segregation analysis	Mother heterozygous for c.3208G>A; father heterozygous for c.4495_4496del
5 months	Outpatient pulmonary follow‐up	Weight 4.45 kg; stable on hydroxychloroquine, prednisone, and azithromycin; baseline tachypnea persisted
6 months, 9 days	Specialist follow‐up	GI and cardiology follow‐up documented; LVH remained stable, and antihypertensives were de‐escalated

### 2.5 Diagnostic Assessment

#### 2.5.1 Diagnostic Methods

Initial chest radiography demonstrated hyperinflation with bilateral infiltrates (Figure 1). An unrevealing upper gastrointestinal study and abdominal ultrasound ruled out pyloric stenosis and gastric outlet obstruction, identifying Grade 3 gastroesophageal reflux with aspiration.

**FIGURE 1:**
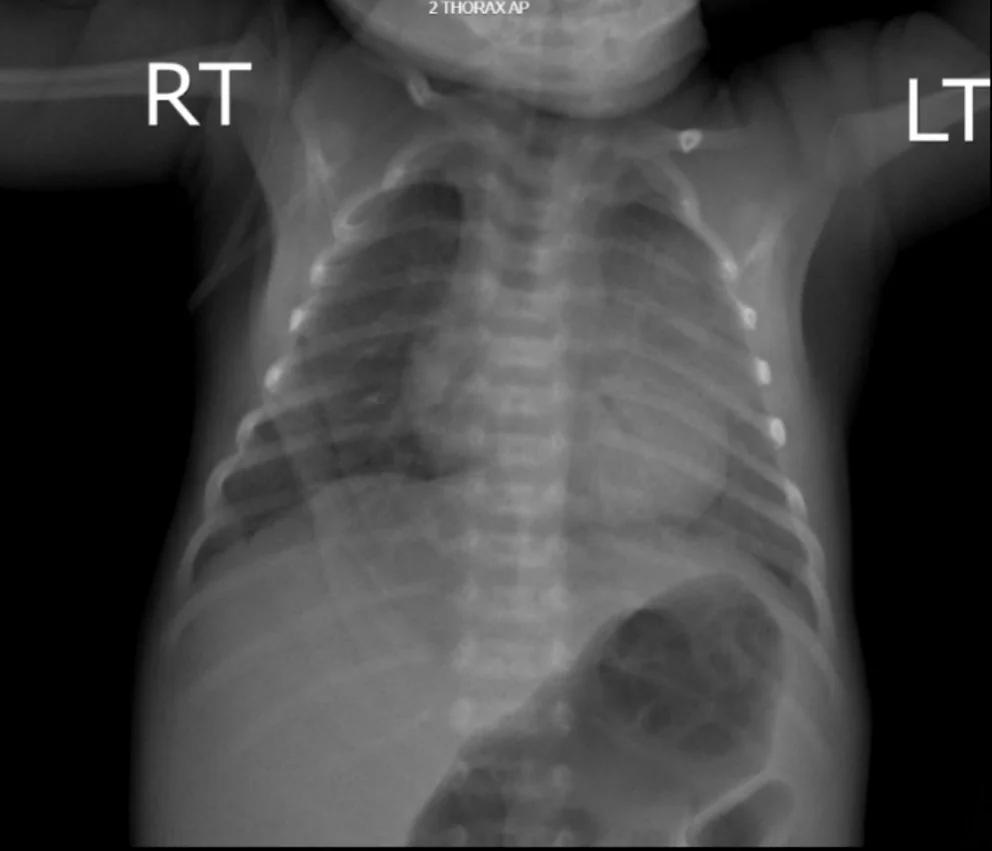
Initial anteroposterior chest radiograph demonstrating bilateral diffuse pulmonary opacities with hyperinflation, prompting further evaluation for an underlying diffuse lung disease.

A chest computed tomography (CT) scan with intravenous contrast revealed bilateral diffuse perihilar consolidation and airspace opacities sparing the apices and basal regions (Figure 2), which remained unchanged on follow‐up high‐resolution CT. A sagittal contrast‐enhanced reconstruction further demonstrated the persistent bilateral pulmonary parenchymal abnormalities and diffuse air‐space opacities, supporting the chronic nature of the disease (Figure 3). A rigid bronchoscopy showed a patent airway with no H‐type tracheoesophageal fistula. Esophagogastroduodenoscopy was grossly normal.

**FIGURE 2:**
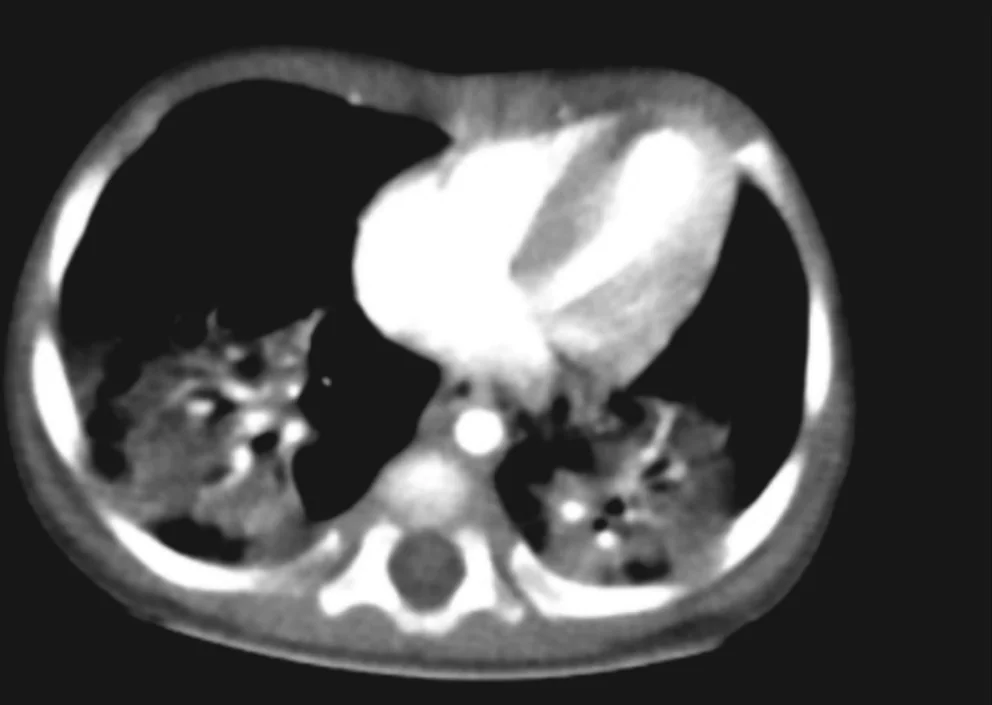
Axial chest CT image demonstrating bilateral diffuse perihilar and posterior lower‐lobe consolidative/air‐space opacities with relative sparing of the peripheral upper lungs, compatible with diffuse interstitial lung disease associated with ABCA3 deficiency.

**FIGURE 3:**
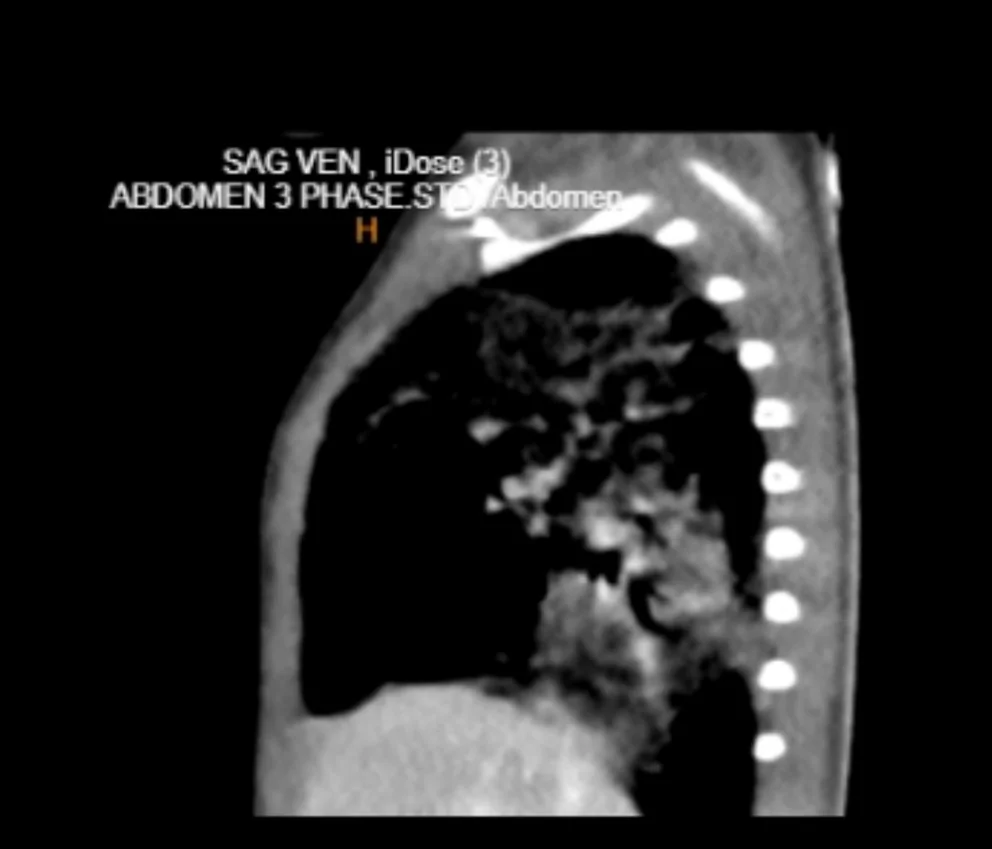
Sagittal contrast‐enhanced chest CT reconstruction demonstrating persistent bilateral pulmonary parenchymal disease with diffuse air‐space opacities predominantly involving the perihilar and lower lung regions, consistent with chronic surfactant dysfunction.

Echocardiography performed at three months of age for new‐onset systemic hypertension revealed a patent foramen ovale, mild tricuspid regurgitation, moderate left‐ventricular hypertrophy, and preserved systolic function with an ejection fraction of 76%. No echocardiographic diagnosis of pulmonary hypertension was documented. Renal CT angiography was unremarkable, providing no evidence of a renovascular cause. A summary of all diagnostic investigations and their clinical significance is presented in Table 2.

**TABLE 2:** Summary of diagnostic investigations, major findings, and their contribution to the final diagnosis.

Investigation	Key result	Clinical significance
Initial CBC and inflammatory markers during early hospitalization	Hgb 15 g/dL, WBC 24.7 × 10^9^/L, platelets 623 × 10^9^/L, CRP negative	Supported acute evaluation but did not establish a unifying cause
Early chest radiography	Left upper‐lobe infiltrate/opacification during neonatal readmission	Prompted respiratory support and antibiotic treatment
PICU chest radiography	Hyperinflation with bilateral infiltrates	Demonstrated a diffuse process rather than isolated focal disease
Chest CT with IV contrast	Bilateral diffuse perihilar consolidation/air‐space opacity sparing apices and basal regions	Suggested diffuse lung disease; structurally helped exclude sequestration
Follow‐up HRCT	No significant interval change	Persistence despite treatment raised suspicion for non‐infectious chronic lung disease
Upper GI contrast study	Grade 3 reflux with aspiration; no gastric outlet obstruction	Established aspiration risk and justified anti‐reflux treatment
Abdominal ultrasound	No pyloric stenosis or major abdominal pathology	Reduced likelihood of common mechanical causes of vomiting
Rigid bronchoscopy	Patent airway; no H‐type TEF	Excluded a key structural mimic
Esophagogastroduodenoscopy	Grossly normal	No major mucosal explanation for symptoms
Immunoglobulin profile	IgA 25, IgE 3, IgM 129, IgG 504	Did not support major humoral immunodeficiency
Echocardiography	PFO, mild tricuspid regurgitation, moderate LVH, preserved EF of 76%; no documented pulmonary hypertension	Supported evaluation of systemic hypertension and documented target‐organ cardiac involvement, but did not identify a primary cardiac explanation for the chronic respiratory disease.
Renal CT angiogram	Unremarkable	Did not support renovascular hypertension
Thyroid profile	TSH 2.52, free T4 1.12	No obvious thyroid explanation for hypertension
Doppler ultrasound of lower limb	Short‐segment DVT at the femoral line site	Identified catheter‐associated thrombosis
Whole‐exome sequencing	ABCA3 c.4495_4496del (*p*.Leu1499AlafsTer20) and c.3208G>A (*p*.Ala1070Thr), both likely pathogenic	Established molecular diagnosis of pulmonary surfactant metabolism dysfunction Type 3
Parental segregation analysis	Mother heterozygous for c.3208G>A; father heterozygous for c.4495_4496del	Confirmed compound heterozygosity in trans

#### 2.5.2 Diagnosis

Whole Exome Sequencing (WES) identified compound heterozygous pathogenic variants in the ABCA3 gene: a frameshift variant (c.4495_4496del, p.Leu1499AlafsTer20) and a missense variant (c.3208G>A, p.Ala1070Thr). Subsequent familial segregation analysis confirmed the mother was heterozygous for the c.3208G>A variant, and the father was heterozygous for the c.4495_4496del variant. These findings confirmed a diagnosis of pulmonary surfactant metabolism dysfunction Type 3. Secondary diagnoses included systemic arterial hypertension with moderate left‐ventricular hypertrophy and Grade 3 gastroesophageal reflux disease with aspiration. The differential diagnostic considerations and their exclusion are summarized in Table 3.

**TABLE 3:** Differential diagnosis considered during the work‐up of recurrent respiratory distress and chronic diffuse lung disease in early infancy.

Differential diagnosis	Why it was considered	Findings arguing against or limiting diagnosis	Final status
Acute bronchiolitis	Cough, rhinorrhea, respiratory distress, initial outside‐hospital labeling	Respiratory disease had been present since birth, persisted beyond the usual bronchiolitis course, and required repeated admissions	Not primary diagnosis
Bacterial pneumonia or recurrent chest infection	Infiltrates on imaging; intermittent clinical deterioration; antibiotics repeatedly used	Persistent abnormalities despite multiple antibiotic courses; cultures frequently negative; later genetic diagnosis explained chronic baseline disease	Intercurrent consideration only
Aspiration‐related lung disease	Vomiting, poor feeding, Grade 3 reflux with aspiration on upper GI study	Important comorbidity, but did not fully explain respiratory distress from birth or genetic findings	Contributory comorbidity
H‐type tracheoesophageal fistula	Suspected because of aspiration history and recurrent respiratory symptoms	Rigid bronchoscopy did not identify a fistula	Excluded
Primary ciliary dyskinesia	Unusual prolonged respiratory course from birth	The empiric steroid/bronchodilator/budesonide trial produced minimal benefit; no confirmatory diagnostic evidence was presented	Unconfirmed; not supported
Cystic fibrosis	Chronic/recurrent respiratory manifestations and poor growth	WES ultimately supported ABCA3‐related disease; no CF‐confirmatory data were reported	Unlikely
Immunodeficiency	Recurrent infection‐like course	Immunoglobulin levels were reported as normal	Less likely
Congenital lung anomaly or sequestration	Persistent CT abnormalities prompted structural evaluation	Chest CT with IV contrast reportedly excluded sequestration	Excluded
Cardiac cause of respiratory distress	Echocardiography performed because of recurrent symptoms and later hypertension	PFO and mild tricuspid regurgitation were insufficient to explain the respiratory phenotype; systolic function was preserved, and no pulmonary hypertension was documented.	Not primary diagnosis
Surfactant metabolism dysfunction	Respiratory distress from birth, persistent oxygen need, chronic infiltrates, negative structural work‐up	Confirmed by WES and parental segregation	Final diagnosis

### 2.6 Therapeutic Intervention

Respiratory support throughout the hospitalization consisted of intermittent NIPPV alternating with nasal cannula oxygen. Prior to genetic diagnosis, an empirical five‐day trial of intravenous methylprednisolone, albuterol, and budesonide for suspected primary ciliary dyskinesia yielded minimal improvement. Following the genetic diagnosis, targeted pharmacotherapy was initiated, comprising azithromycin, systemic prednisone, and hydroxychloroquine.

Gastrointestinal management included esomeprazole (1 mg/kg twice daily), metoclopramide, and a transition to an amino acid‐based formula (Neocate) with medium‐chain triglyceride oil supplementation. At four months of age, due to persistent feeding difficulties and suspected obstruction, the patient underwent an exploratory laparotomy. Intraoperatively, adhesions causing kinking at the fourth part of the duodenum and proximal jejunum were released, and an orojejunal tube was inserted.

Systemic hypertension was managed with a step‐up multidrug regimen including captopril, propranolol, atenolol, and amlodipine, with intravenous labetalol utilized for acute stabilization. Multiple courses of intravenous piperacillin‐tazobactam were administered for suspected clinical sepsis and a central line‐associated *Staphylococcus epidermidis* bloodstream infection. The complete therapeutic course and treatment timeline are summarized in Table 4.

**TABLE 4:** Therapeutic timeline showing respiratory support, empiric pharmacologic therapy, targeted post‐genetic therapy, gastrointestinal interventions, hypertension management, and treatment of line‐related complications.

Period	Intervention	Rationale	Observed response
Neonatal period	Oxygen support and NICU care	TTN‐like respiratory distress/desaturation	Partial short‐term stabilization
Age 11 days admission	NIPPV for 3 days plus ampicillin and cefotaxime	Recurrent respiratory distress with left upper‐lobe opacity	Improved enough for discharge
PICU admission	NIPPV alternating with nasal cannula oxygen	Escalating acute respiratory failure	Recurrent dependence; difficult weaning
Early prolonged admission	Piperacillin‐tazobactam courses	Suspected pneumonia/clinical sepsis	No definitive resolution of underlying disease
Suspected PCD phase	5‐day IV methylprednisolone, albuterol, budesonide	Trial for alternate inflammatory/ciliary diagnosis	Minimal improvement
After reflux evaluation	Esomeprazole and metoclopramide; AR then Neocate formula	Grade 3 reflux with aspiration and feeding intolerance	Partial GI support, but vomiting persisted
After WES diagnosis	Azithromycin 10 mg/kg/day, prednisone/prednisolone, hydroxychloroquine 5 mg/kg/day	Empiric targeted therapy for ABCA3‐related chronic/intermediate phenotype	Eventual outpatient stabilization; baseline tachypnea persisted
Hypertension onset	IV labetalol as needed, then captopril, propranolol, atenolol, amlodipine	Secondary systemic hypertension with LVH	Subsequent improvement; later weaned to captopril monotherapy
Feeding/obstruction phase	MCT oil supplementation and later exploratory laparotomy with adhesiolysis and orojejunal tube insertion	Poor weight gain, persistent vomiting, suspected obstructive component	Enteral tolerance improved after operative management
Catheter‐associated DVT	Enoxaparin 1 mg/kg every 12 h	Right lower‐limb swelling with Doppler‐confirmed DVT	Anticoagulation continued with vascular follow‐up
Outpatient prevention	Chest physiotherapy, planned palivizumab, influenza vaccine, avoidance of live vaccines	Reduce viral burden and secretion retention during chronic lung disease follow‐up	Part of the long‐term supportive plan

### 2.7 Follow‐Up and Outcomes

By five months of age, the patient was managed in the outpatient setting. She weighed 4.45 kg, demonstrating acceptable interval weight gain. Respiratory status was maintained on the targeted triplet regimen (hydroxychloroquine, prednisone, and azithromycin). Clinical examination revealed baseline respiratory distress characterized by tachypnea and subcostal retractions, alongside minimal stridor associated with exertion, attributed to laryngomalacia. Air entry remained good bilaterally.

Cardiac follow‐up demonstrated stable moderate left‐ventricular hypertrophy without documented pulmonary hypertension. Following improvement in systemic arterial blood pressure, the multidrug antihypertensive regimen was successfully reduced to captopril monotherapy, without any required cardiac restrictions. Preventative care plans included palivizumab administration, seasonal influenza vaccination, and daily chest physiotherapy, with strict avoidance of live‐attenuated vaccines. No adverse drug events or unmanageable complications from the targeted therapy were reported during the follow‐up period.

## 3 Discussion

This infant's disease course is most consistent with an intermediate ABCA3 phenotype. The lecture material provided with the case highlights a clinically useful genotype framework: biallelic null variants are usually associated with fulminant neonatal respiratory distress, whereas combinations that preserve partial residual function may present with persistent oxygen requirement and infantile chronic interstitial lung disease rather than immediate fatal collapse. In the present patient, the novel paternal frameshift c.4495_4496del is a loss‐of‐function allele, while the maternally inherited p.Ala1070Thr missense variant has prior association with interstitial lung disease. That combination provides a biologically plausible explanation for severe but non‐fatal early disease with survival into outpatient follow‐up [2, 3].

A major lesson from this case is the diagnostic cost of non‐specific early framing. Term infants with ABCA3 deficiency are often initially labeled as transient tachypnea, bronchiolitis, recurrent pneumonia, or aspiration‐related disease because their presentation overlaps these common disorders. In this infant, the combination of respiratory distress from birth, repeated admissions, failure to remain off respiratory support, persistent radiologic abnormalities despite antibiotics, and non‐diagnostic bronchoscopy should lower the threshold for surfactant‐dysfunction testing. The extensive differential pursued here was clinically appropriate, but the persistence of disease beyond typical bronchiolitis and the absence of a correctable structural lesion were particularly strong signals that a genetic diffuse lung disease was more likely [4].

The radiologic pattern also deserves emphasis. The chest CT showed bilateral diffuse perihilar consolidation/air‐space opacity with relative apical and basal sparing, and this remained unchanged on follow‐up high‐resolution CT (Figure 4), despite anti‐infective treatment. Although classically described surfactant‐dysfunction imaging often emphasizes diffuse or ground‐glass interstitial change, real‐world ABCA3 disease is radiologically heterogeneous, especially during acute infantile decompensation. Accordingly, the CT images were used primarily to demonstrate the distribution and persistence of the pulmonary abnormalities and to exclude major structural abnormalities, rather than for detailed phenotypic characterization or etiologic confirmation. Molecular testing ultimately provided the definitive diagnosis [5, 6].

**FIGURE 4:**
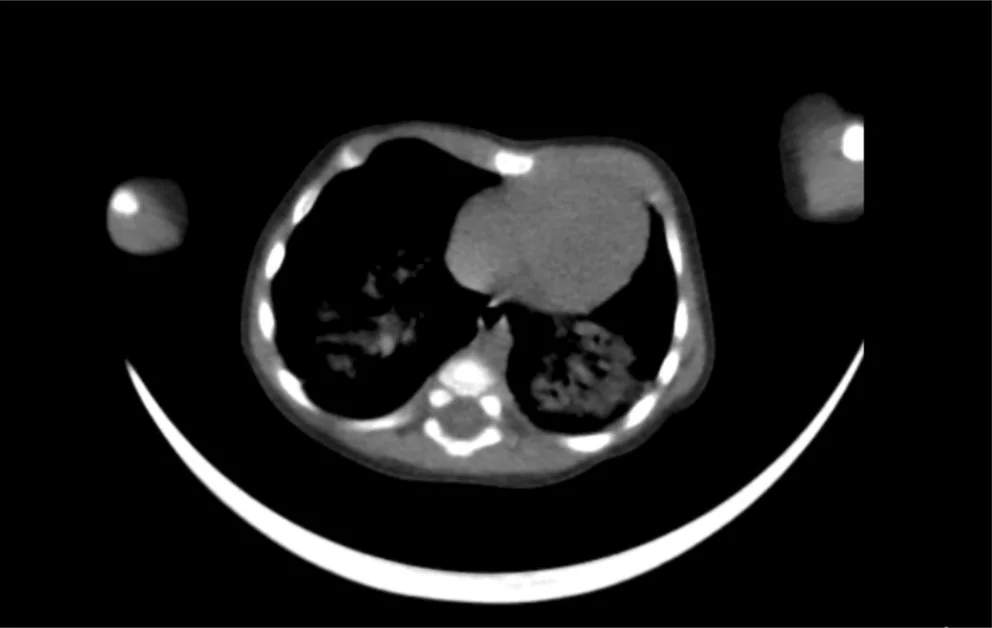
Axial chest CT image obtained at a different level showing persistent bilateral air‐space opacities without focal congenital structural lung abnormalities, supporting diffuse pulmonary surfactant metabolism dysfunction.

Management was necessarily multidisciplinary and pragmatic. The lecture file supplied with the case summarizes current practice in ABCA3‐related chronic/intermediate phenotypes as largely supportive, sometimes supplemented by empiric trials of hydroxychloroquine, systemic corticosteroids, and azithromycin because definitive treatment trials remain limited and responses are heterogeneous. In the present infant, the introduction of azithromycin, prednisone, and hydroxychloroquine after molecular confirmation coincided with eventual outpatient stabilization, acceptable interval weight gain, and no documented major adverse drug events during the available follow‐up. Even so, the patient remained tachypneic at baseline, which underscores that stabilization should not be mistaken for cure and that serial reassessment of oxygen requirement, growth, infection burden, ophthalmologic safety, and cardiopulmonary status remains essential [7, 8].

The cardiovascular findings require careful interpretation. Pulmonary hypertension has been described in patients with ABCA3 deficiency, including persistent pulmonary hypertension of the newborn and pulmonary hypertension complicating progressive interstitial lung disease or pulmonary fibrosis. However, the hypertension documented in our patient was systemic arterial hypertension accompanied by left ventricular hypertrophy, rather than echocardiographically confirmed pulmonary hypertension [9, 10]. Therefore, the available evidence does not establish a direct causal relationship between the ABCA3 variants and her systemic hypertension. Renovascular imaging was unremarkable, and the hypertension was considered a secondary, potentially multifactorial comorbidity requiring independent evaluation and treatment. Other complications, including reflux with aspiration, poor weight gain, catheter‐associated thrombosis, and recurrent suspected sepsis, further increased her respiratory vulnerability and complicated clinical management [11, 12].

The case therefore argues for a broad but disciplined chronic‐lung‐disease pathway in ABCA3 deficiency, one that integrates pulmonary care with cardiology, gastroenterology, surgery, nutrition, physiotherapy, genetics, and infection prevention. Lung transplantation must remain part of longitudinal counseling should progression outpace supportive and empiric medical therapy.

## 4 Conclusion

This case demonstrates that ABCA3‐related pulmonary surfactant metabolism dysfunction Type 3 should be strongly considered in any term infant with respiratory distress from birth, recurrent “bronchiolitis/pneumonia” admissions, persistent oxygen requirement, and non‐resolving diffuse lung infiltrates after common structural, infectious, and aspiration‐related causes have been excluded. Whole‐exome sequencing with parental segregation analysis was diagnostic and clinically transformative in this patient, allowing the disease to be reclassified from recurrent infection to inherited surfactant dysfunction. The compound heterozygous genotype, consisting of a novel frameshift allele and a missense allele previously linked to interstitial lung disease, is concordant with an intermediate phenotype. Although pulmonary hypertension has been reported in association with ABCA3 deficiency, the systemic arterial hypertension observed in our patient cannot currently be considered a recognized manifestation of the disorder and requires independent evaluation and management. Short‐term stabilization on azithromycin, prednisone, and hydroxychloroquine was encouraging, but continued multidisciplinary surveillance remains essential.

## Author Contributions

**Ammir Abuzahra:** writing – original draft, conceptualization, methodology, writing – review and editing. **Mohamad Abu Mayalah:** data curation, project administration. **Mahmoud Abdelrazzaq Abu Mayaleh:** writing – original draft, methodology, software. **Amani Daabes:** resources, validation. **Rafad Y. Dweik:** visualization, investigation. **Abdelrazzaq Abu Mayaleh:** supervision.

## Funding

The authors have nothing to report.

## Disclosure

The authors have nothing to report.

## Consent

Written informed consent for publication of the clinical details and any accompanying images was obtained from the patient's legal guardians before submission.

## Conflicts of Interest

The authors declare no conflicts of interest.

## Data Availability

The data that support the findings of this study are available on request from the corresponding author. The data are not publicly available due to privacy or ethical restrictions.
